# A pilot multimodal study of cervical cancer: Raman spectroscopy as a molecular fingerprint tool

**DOI:** 10.1371/journal.pone.0327286

**Published:** 2026-01-22

**Authors:** Anacleto Proietti, Emanuele De Angelis, Luca Buccini, Martina Leopizzi, Angelina Pernazza, Francesco Mura, Angelica Accorinti, Gianluca Sbardella, Giancarlo La Penna, Roberta Maria Arseni, Giorgia Perniola, Daniele Passeri, Carlo Della Rocca, Marco Rossi, Violante Di Donato

**Affiliations:** 1 Department of Basic and Applied Sciences for Engineering, Sapienza University of Rome, Rome, Italy; 2 Department of Obstetrics and Gynecology, University Sapienza of Roma, Rome, Italy; 3 Department of Medico-Surgical Sciences and Biotechnology, Polo Pontino, Sapienza University of Roma, Latin, Italy; 4 Department of Pathology, Umbert I Hospital, Rome, Italy; 5 Research Center for Nanotechnology Applied to Engineering of Sapienza University of Rome (CNIS), Rome, Italy; IIIT Kurnool: Indian Institute of Information Technology Design and Manufacturing Kurnool, INDIA

## Abstract

Cervical cancer remains a significant global health burden, highlighting the need for more effective tools for early detection and tissue characterization. In this study, we propose a multimodal strategy that combines Raman spectroscopy, Atomic Force Microscopy (AFM), and Scanning Electron Microscopy (SEM) to investigate the molecular and morphological features of cervical squamous cell carcinoma (SCC). Raman spectroscopy was used to analyze biochemical signatures across different tissue regions tumor, necrotic, stromal, and glandular within the 813–1668 *cm*^*-1*^ range, identifying distinct molecular profiles between malignant and healthy areas. Specific vibrational peaks associated with DNA, proteins, and lipids were examined to track molecular changes related to tumor progression. AFM enabled nanoscale mapping of surface morphology, revealing structural irregularities associated with malignancy, while SEM provided detailed imaging of cellular and extracellular architecture, enhancing the visualization of cancer-induced morphological alterations. Although Raman spectroscopy has been studied for decades in cancer research, it has not yet replaced Pap smears and biopsies in clinical practice due to challenges in standardization, reproducibility, and clinical validation. This pilot study aims to serve as a stepping stone toward that goal, providing proof-of-concept data that may support the gradual translation of Raman spectroscopy into clinically relevant diagnostic workflows and underscores the potential of the technique, supported by complementary high-resolution imaging techniques, in the characterization of cervical cancer tissues. The integration of Raman, AFM, and SEM was used here as a pilot approach on paraffin-embedded samples, with AFM and SEM providing supportive morphological information while the long-term aim is to transfer Raman spectroscopy to fresh, untreated tissues, where its non-destructive and label-free nature could enable minimally invasive diagnostic applications.

## 1. Introduction

Cervical cancer is the fourth most common cancer among women worldwide, with 660,000 new cases and 350,000 deaths reported in 2022 [1]. The burden of the disease is particularly high in low-resource settings due to limited access to screening and preventive measures [2]. Cervical cancer develops progressively from precancerous lesions, classified as low-grade (LSIL) or high-grade (HSIL) squamous intraepithelial lesions, with HSIL often progressing to squamous cell carcinoma (SCC) [3]. High-risk human papillomavirus (HPV) infections, primarily HPV-16, −18, −31, and −33, are the leading etiological factors [4]. Oncoproteins E6 and E7 produced by HPV disrupt cell cycle control, promote uncontrolled proliferation, and inhibit apoptosis, driving malignant transformation [5–6].

Early detection and accurate characterization of cervical cancer can significantly reduce mortality through timely intervention. Current diagnostic methods rely primarily on cytology, histopathology, and HPV testing, but these approaches have limitations, including interobserver variability and the need for invasive biopsies [7–8]. Raman spectroscopy has emerged as a promising tool for cancer diagnostics by providing detailed molecular fingerprints of tissues with minimal sample preparation [9–10]. In cervical cancer, Raman-based analysis has demonstrated the ability to distinguish malignant from healthy tissues based on characteristic biochemical signatures [11–14] based, i.e., on differences in structural proteins like collagen and amides, as reflected in peaks at 813 *cm ⁻ ¹* and 827 *cm ⁻ ¹* [15–16].

Although Pap smear cytology and histopathological examination currently represent the gold standard for cervical cancer screening and diagnosis, these conventional approaches can be limited by sample preparation requirements, interobserver variability, and their inability to provide detailed molecular information. In this context, Raman spectroscopy emerges as a non-destructive, label-free, and highly sensitive analytical technique capable of detecting subtle biochemical alterations at the molecular level, often preceding visible morphological changes. Its ability to differentiate nucleic acids, proteins, and lipids within complex tissue environments makes it an attractive tool for oncological diagnostics.

Raman spectra of preserved cervical tissue biopsies have shown potential in distinguishing pre-cancerous from normal tissues, achieving sensitivity and specificity around 90% [17].

Principal component analysis applied to these spectra yielded high sensitivity and specificity of up to 99.5% [18] and automated Raman spectroscopy systems have also been proposed for high-throughput screening in real time.

Despite decades of research, Raman spectroscopy has not yet supplanted Pap smears or biopsies in the diagnostic workflow for cervical cancer. The main barriers include the complexity of spectral interpretation, variability arising from tissue preparation methods, and the lack of large-scale clinical validation. Overcoming these limitations requires carefully designed pilot studies that demonstrate both feasibility and reproducibility. The present work is conceived as such a stepping stone, with the long-term goal of enabling Raman spectroscopy to move from experimental investigations toward integration into clinical diagnostics.

Raman spectroscopy provides biochemical information [19–20] that makes it a promising candidate for future diagnostic applications. In this study, AFM and SEM were then employed as complementary tools to provide nanoscale and microscale morphological context. The aim of this pilot study is therefore to explore the potential of Raman spectroscopy to characterize cervical carcinoma tissue and to identify spectral biomarkers capable of distinguishing healthy from malignant regions. This was performed on paraffin-embedded samples, acknowledging the limitations of FFPE (Formalin-Fixed, Paraffin-Embedded) processing, while paving the way for future applications on fresh, untreated tissues where Raman could be applied in a clinically relevant diagnostic setting. The integration of AFM and SEM in this work should not be interpreted as an attempt to design a multimodal diagnostic workflow, since these techniques are not compatible with the time constraints of clinical practice. Rather, their role in this pilot study is to provide supportive micro- and nanoscale morphological information that strengthens the interpretation of Raman spectra. By directly correlating biochemical signatures with structural features beyond the resolution of conventional optical microscopy, AFM and SEM serve as a methodological foundation for Raman analysis. This helps to address one of the key limitations in the field, namely the lack of robust morphological correlations that has hindered the standardization and clinical adoption of Raman spectroscopy. Ultimately, Raman spectroscopy remains the central diagnostic candidate, and future studies will focus on applying it independently supported by statistical tools such as PCA and LDA on larger cohorts and fresh tissues, where its rapid and non-invasive nature may be fully leveraged for clinical use.

## 2. Methods

This study was conducted under the approval of the Institutional Ethics Committee of Sapienza University of Rome (Ref. 7896, Prot. 0187/2025). The analysis was performed exclusively on archived paraffin-embedded tissue samples, and no experimentation was conducted on living human subjects. Two Cervical Squamous Cell Carcinoma cases were selected from the archives of the Pathology Department (Sapienza, University of Rome – Umberto I Hospital). For the analysis the samples were fixed in 10% neutral buffered formalin for 48-72h. For paraffin embedding, fixed specimens were dehydrated using graded alcohols (50%−70%−95%−100%) to remove water, cleared in xylene and processed in a tissue processor under vacuum conditions (Tissue-Tek VIP 5 Jr., Sakura Finetek Japan Co. Ltd., Japan) and then embedded in paraffin wax. To the study purposes, the cases were reviewed by the pathologist who chose the two most representative paraffin tissue blocks containing the tumor area and a counterpart of normal tissue. The tissue blocks were sliced into 3 *µm* serial sections and mounted on Bio Microscope Slide (Bio-Optica S.p.a.-Milan-Italy composed of soda-lime glass). The optical images on the stained samples (stained with hematoxylin and eosin) were then acquired with VENTANA DP 200 slide scanner (Roche, Basel, Switzerland), S1 Fig.

After the optical analysis the adjacent unstained slides (3 *µm* before the stained ones) dedicated to Raman, SEM and AFM analysis were not stained (to avoid spectral artifacts related to the staining), immediately deparaffinated (two changes of xylene at 15 minutes each) and dried to the air.

Subsequently Scanning Electron Microscopy (SEM), Atomic Force Microscopy (AFM) and Raman Spectroscopy were used to identify morphological characteristics and to acquire molecular profiles with the final goal to create a baseline that will allow to identify specific spectral markers capable of distinguishing healthy from malignant areas.

It should be noted that the adopted procedure is adequate to avoid artifacts in this type of analysis. However, the main objective of the present work was to demonstrate the feasibility of the multimodal approach. The long-term goal is to develop a protocol that can be applied without any prior sample preparation.

### 2.1. Scanning electron microscopy

SEM micrographs were obtained using FESEM Zeiss Auriga 405. For the imaging, a conductive coating of chromium with a thickness of 10 *nm* has been deposited on the same surface used for the other analysis using a Quorum Q150T sputter machine and without needs for any other type of preparations.

### 2.2. Atomic force microscopy

Atomic Force Microscopy measurements, 50 × 50 *μ*m at 512 points/line, were performed using Dimension ICON AFM (Bruker) equipped with a NGS probe (NT-MDT) in tapping mode in every area of interest without any need for sample preparation after the optical imaging. All the scans were processed using Gwyddion (software version 2.66).

### 2.3. Raman spectroscopy

Raman spectra were collected using a confocal inVia^TM^ Raman Spectrometer (from Renishaw), with 250 *mm* focal length. The specimens were analyzed at room temperature in the 180−1940 *cm*^*-1*^ spectral range. The signal is dispersed by a holographic grating of 1800 *lines/mm* and collected by a Peltier-cooled CCD detector. The laser source used to excite the sample is manufactured by Renishaw and have a wavelength of 532.1 *nm* with an output power of 50 *mW*.

The laser beam was focused onto the sample through a 100x N-Plan objective (NA = 0.88, WD = 0.33 *mm*) and the theoretical laser spot size (Airy disk diameter) on the sample was estimated at ~0.74 *μm*, calculated using the relation d = 1.22 λ/ NA. The acquired Raman spectra were processed using the software WiRE^TM^ 4.4 to remove the cosmic rays, normalize the spectra and remove the baseline. The laser power impinging on the samples was set to 25 *mW* with an exposure time of 1 *s* and 10 accumulations. The peak positions were calibrated using both an internal and an external silicon reference. Baseline correction was performed using an adaptive polynomial algorithm (window size = 3, polynomial order = 11) to minimize fluorescence background, while normalization approaches were applied contextually depending on the specific spectral comparison. Each Raman scan was acquired from a different analysis point, with variable spacing between measurement sites. A minimum spacing of 10 *µm* was maintained to avoid spectral overlap and to ensure significance of the collected data. The intensity (peak height) and position of the main Raman bands were subsequently analyzed to evaluate spectral variations among the different tissue regions.

## 3. Results

### 3.1. Histological analysis

**Fig 1:**
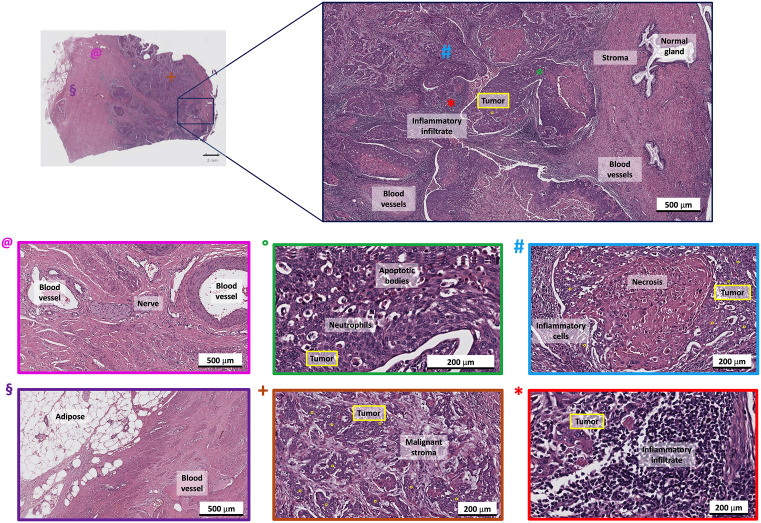
Histological analysis of the first slide. Representative regions were identified by the pathologist, including healthy areas (glands, stroma, nerves, blood vessels) and tumor-associated areas (tumor nests, necrosis, inflammatory infiltrate, malignant stroma). The colored boxes indicate the corresponding regions from which Raman spectra were subsequently acquired, ensuring that both healthy and malignant tissues were analyzed.

Histological analysis of the cervical cancer specimen revealed complex tissue architecture and tumor microenvironment, as shown in Fig 1.

Pink-dot regions indicated nerves and blood vessels, while green-dot areas showed adipose tissue with vacuolated cells characteristic of lipid depletion. Green-dot regions also displayed apoptotic bodies with fragmented nuclei and neutrophils identified by their multi-lobed nuclei. The orange-dot areas highlighted malignant stroma with densely packed fibrous tissue and a disrupted extracellular matrix consistent with desmoplasia. Blue-dot regions exhibited necrotic tissue, marked by acellular eosinophilic debris and surrounded by inflammatory cells.

Malignant cells exhibited hallmark features of squamous cell carcinoma, including pleomorphic appearance, hyperchromatic nuclei, and a high nucleus-to-cytoplasm ratio. The dense inflammatory infiltrate, primarily lymphocytes and macrophages, underscored an immune response to tumor antigens, although potential immune evasion mechanisms were noted. Imaging of the entire section was also performed at this stage (S1 Fig) to ensure that the same areas of interest were analyzed through all the techniques used.

### 3.2. Scanning electron microscopy

The scanning Electron Microscopy images provide detailed insights into the microstructural differences in cervical squamous cell carcinoma and surrounding tissues.

**Fig 2:**
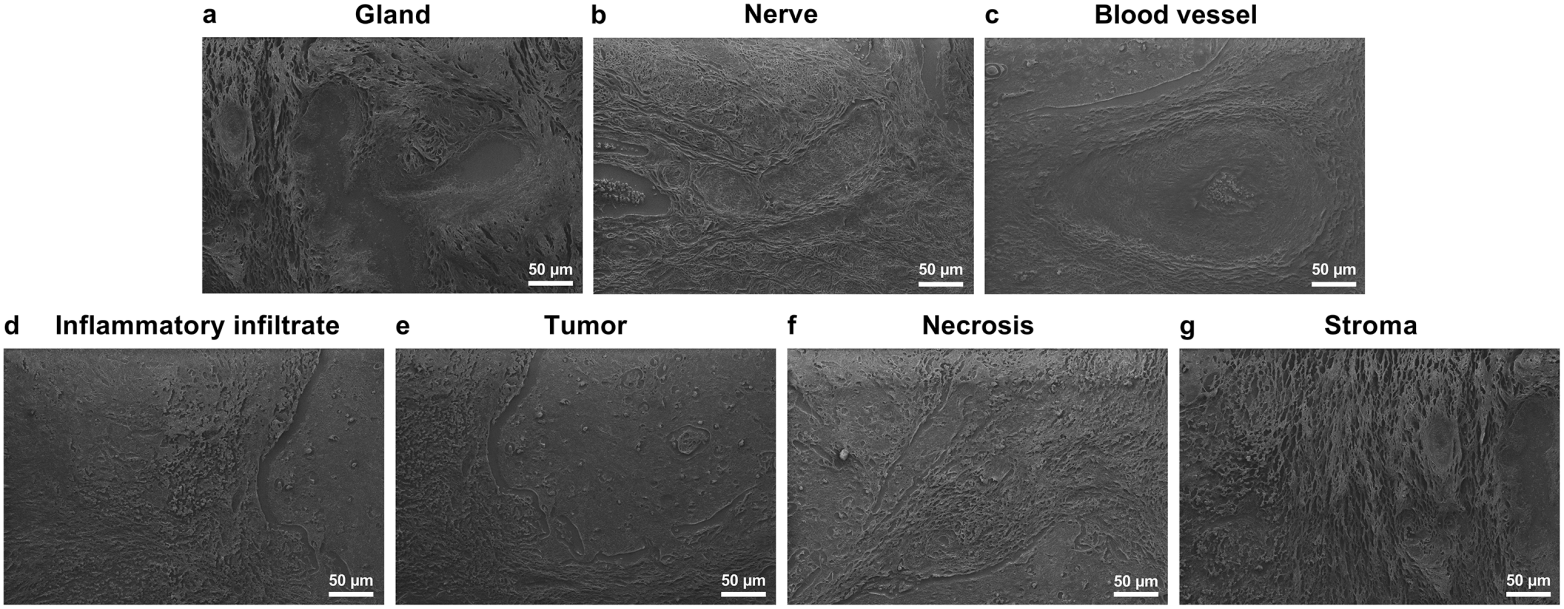
Scanning electron microscopy images of (a) gland, (b) nerve, (c) central region of a blood, (d) inflammatory infiltrates, (e) tumor, (f) necrotic tissue and (g) stroma.

Fig 2-a shows a gland with a compact cellular arrangement, distinguishing it from surrounding tissues [21]. Fig 2-b depicts a nerve composed of intertwined fibers, likely axons bundled by supportive tissues [22]. Fig 2-c focuses on a blood vessel with a central structure coated in polygonal microparticles, while the outer layer appears compact and porous, resembling the tunica externa (S2 Fig). Inflammatory infiltrates (Fig 2-d) exhibit instead an irregular tissue structures with rounded asperities, reflecting immune cell infiltration and extracellular matrix disorganization during inflammation. Tumor tissue (Fig 2-e) shows reduced porosity and a compact structure, indicating aggressive proliferation and extracellular matrix densification [23,24]. Necrotic tissue (Fig 2-f) displays greater irregularity, with cellular debris and extracellular material covering the surface, indicative of advanced necrosis [25]. Finally, Fig 2-g highlights the stroma structure composed of a fibrous network (S3 Fig) supporting structural integrity and nutrient diffusion, crucial for tissue homeostasis [26].

### 3.3. Atomic force microscopy

The Atomic Force Microscopy images complement the Scanning Electron Microscopy analysis by providing detailed insights into surface roughness and porosity, aiding in the differentiation of pathological changes associated with tumor progression.

**Fig 3:**
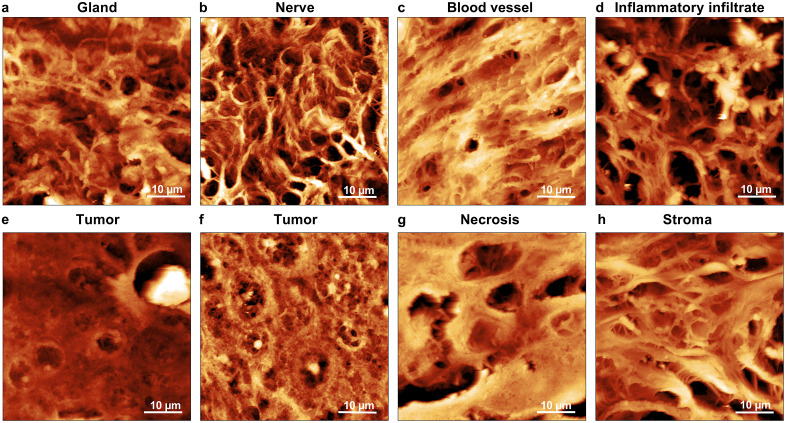
Atomic force microscopy image (a) of a glandular structure, (b) nerve, (c) blood vessel, (d) inflammatory tissue, (e, f) tumor, (g) necrotic tissue and (h) stroma.

Fig 3-a depicts a glandular structure that show both a fibrous and porous structure, while Fig 3-b shows a nerve structure with tightly organized fibers aligned in a manner consistent with axonal bundles and myelin sheaths [27]. Fig 3-c illustrates the structure of the blood vessel, while Fig 3-d highlights inflammatory tissue with an extremely porous and irregular surface most likely composed of extracellular material produced during inflammation, reflecting active tissue remodeling and immune infiltration [28–29]. Figs 3-e and 3-f reveal tumor tissue characterized by reduced porosity and a more compact structure. This compactness indicates dense tumor cell proliferation, with localized heterogeneity in growth and extracellular matrix production to support further expansion [30]. Fig 3-g, depicting necrotic tissue, shows large holes and a uniform matrix coating, likely consisting of cellular debris or proteinaceous material. This layer reflects extensive tissue degradation and the transition to a non-viable state, consistent with advanced necrosis [31]. Finally, Fig 3-h highlights the stroma, displaying a porous fibrous network essential for providing mechanical support and maintaining structural integrity. While the thickening of the structure from inflammatory infiltrate to tumor to necrosis is clearly visible it is seen that it is difficult to recognize the stroma from gland, blood vessels and nerves because of its both porous and fibrous structure.

### 3.4. Raman spectroscopy

After morphological analysis, Raman analysis was performed in the same areas in order to correlate spectral changes with morphological changes confirmed through the other techniques.

Initially, spectra from the microscope slide itself were recorded, showing distinct bands at 470, 574, 788, 952, 996, and 1097 *cm*^*-1*^ (S4 Fig). Since these bands originate from the glass substrate and cannot be completely avoided, they were not subtracted computationally in order to prevent artifacts. Instead, the corresponding spectral regions were masked, and spectra with evident glass contributions were excluded from subsequent tissue analysis to ensure reliable interpretation of Raman signals.

**Fig 4:**
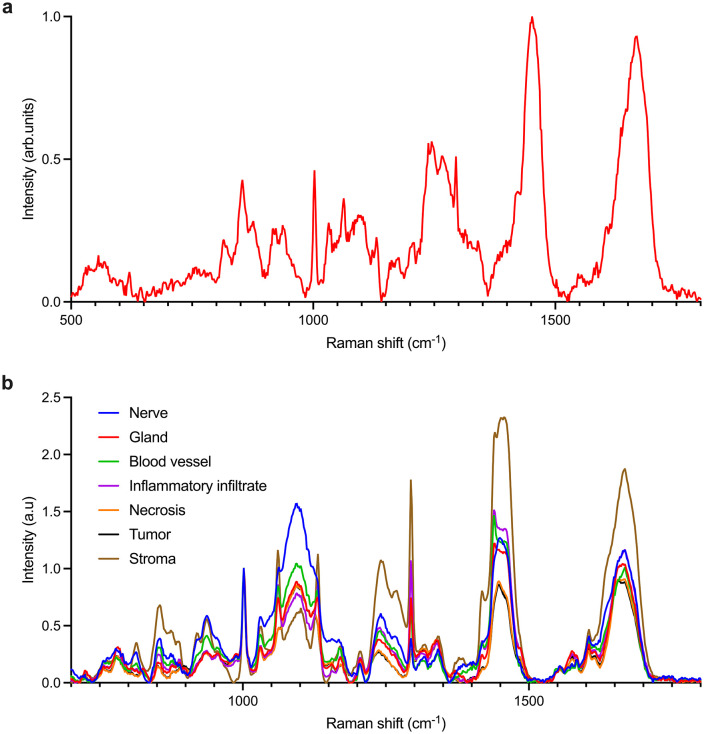
(a) A typical Raman spectrum of one histological section (normalized at the highest intensity peak), (b) overlaid averaged Raman spectra (50 per tissue type) from nerve, gland, blood vessel, inflammatory infiltrate, necrosis, tumor, and stroma normalized to the phenylalanine band at 1003 cm^-1^ to enable the comparison between the spectra. For improved clarity, an alternative visualization of these spectra is provided in S5 Fig of the Supplementary Material.

Initial spectra obtained from these sample in the healthy area on stroma were analyzed and several Raman bands were identified in the spectra (Fig 4-a).

These Raman bands are resumed in Table 1.

**Table 1:** Raman peak assignment of the spectra depicted in Fig 4 [32,33].

Raman shift (*cm*^*-1*^)	Molecular assignment
567	Cholesterol, Phosphatidylinositol
621	Glycerol/ C–C twisting mode, Phenylalanine
754	Tryptophan
817	C–C stretching, Collagen vibrations
855	Proline, Tyrosine, Collagen
877	Lipids
910–970	Protein bands, Lipids
1003	Phenylalanine
1032	Phenylalanine, Collagen
1063	C–C stretch (Lipids); possible paraffin/slide artifacts
1098	C–C stretch, C–C skeletal stretch (trans), PO₂ symmetric vibrations
1120–1150	Protein, Phenylalanine, Lipids
1170	CH in-plane bending of Tyrosine
1200	Antisymmetric stretch of phosphate vibrations
1207–1275	Amide III, Collagen
1290	Cytosine
1314	CH₃/CH₂ twisting or bending (Lipids, Collagen)
1341	C–C stretch of Phenylalanine
1437	CH₂ deformation (Lipids)
1445	CH₃/CH₂ deformation (Lipids)
1506	Cytosine
1510	A ring breathing modes in DNA
1560	Tryptophan
1576	Ring breathing modes of adenine and guanine
1583	Phenylalanine
1618	Tryptophan protein assignment
1655–1680	Amide I (protein folding, structural remodeling)

After that, the same areas analyzed in AFM and SEM were analyzed by Raman spectroscopy enabling differentiation between glands, stroma, tumor, inflammatory infiltrates, necrosis, nerves, and blood vessels.

Within the 813–1668 *cm*^*-1*^ range (Fig 4-b), Raman spectra captured molecular vibrations associated with collagen, nucleic acids, lipids, and the Amide I band of proteins, as detailed in Table 1. Variations in these bands reflect processes such as extracellular matrix remodeling, nucleic acid accumulation, lipid membrane alterations, and protein structural changes during tumor progression (S6 Fig).

The 813 *cm*^*-1*^ peak, prominent in nerve, blood vessel and stromal regions, is attributed to collagen vibrations. Its strong signal correlated with the preservation of structural integrity, supported by SEM and AFM images showing porous yet fibrous and regular architectures typical of collagen-rich tissues.

Reduced intensity at 854–890 *cm*^*-1*^ in tumors and necrotic tissues corresponded to lipid and protein loss, a hallmark of cellular degradation [17].

Increased intensity at 920–956 *cm*^*-1*^ in nerves, stroma, and blood vessel reflected the strong contribution of protein and lipid vibrational modes in these regions, consistent with their role in maintaining structural integrity and supporting active biochemical and signaling processes.

Lower intensities at 1172–1268 *cm*^*-1*^ in tumor and necrotic tissues reflected the reduction of tyrosine, phosphate group vibrations, and Amide III/collagen signals, consistent with protein degradation, nucleic acid alteration, and extracellular matrix disorganization. These molecular changes are aligned with Scanning Electron Microscopy and Atomic Force Microscopy observations of structurally disrupted and disorganized regions.

The change of the position of the peaks at 1655 *cm*^*-1*^ in gland, inflammatory infiltrate, tumors and necrosis marked amide bond accumulation, a sign of malignancy and tissue breakdown.

Differences in nucleic acid signals (1200–1400 *cm*^*-1*^) indicated heightened cellular activity in diseased tissues, while lipid alterations (1450–1600 *cm*^*-1*^) reflected membrane remodeling while protein band variations (1600–1750 *cm*^*-1*^) suggested structural modifications linked to pathological processes.

Following the Raman analysis on the first slide, a second histological section obtained from a different patient was analyzed to evaluate the reproducibility of the results, to assess potential inter-patient variability and to confirm the ability of the technique in differentiating healthy from diseased tissues.

Several Raman spectra were acquired from this second slide (S7 Fig), specifically targeting both tumor-affected areas and regions histologically identified as morphologically normal or unaffected.

The spectral profiles from the second sample revealed high consistency with the patterns observed in the first slide. But, to further investigate the ability of Raman spectroscopy to distinguish between diseased and healthy areas, 200 spectra acquired from different tissue region (tumoral and non-tumoral) were averaged to extract representative spectral fingerprints (S8 Fig). This averaging process reduced local heterogeneities linked to different component of the tissue and enhanced the visibility of more consistent spectral features across regions.

**Fig 5:**
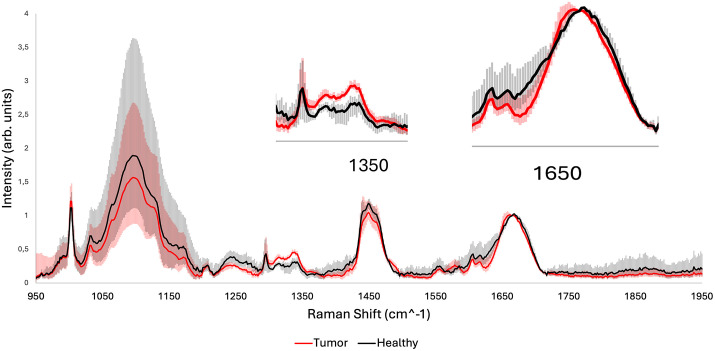
200 healthy and 200 sick averaged Raman spectra from the second slide with their variability (represented as the minimum–maximum range of spectral intensities at each Raman shift). Insets zooms-in on the peaks that show enough variability to allow recognition between the spectra of tumor tissue and healthy tissue after baseline removal normalized to the phenylalanine band at 1650 cm^-1^.

Notably, two key peaks at approximately 1341 *cm*^*-1*^ and 1650 *cm*^*-1*^ emerged as possible reliable indicators of disease state as can be seen from Fig 5. The peak at 1341 *cm*^*-1*^ is generally associated with nucleic acid content and was consistently elevated in tumor regions, reflecting increased DNA and RNA activity, while the peak at 1650 *cm*^*-1*^ linked to the Amide I band and protein conformational changes was intensified in neoplastic zones, confirming the presence of necrosis or tumor hence confirming the robustness of the Raman spectroscopy for identifying these areas at a macro-level.

Overall, the combined analysis confirms that the Raman spectral features used to differentiate tissue types are not only reproducible across technical replicates but also consistent across two biological replicates from different patients (despite this is true only for this two case studies, a larger cohort of samples and patients is needed to prove this statement).

Furthermore we acknowledge that formalin fixation and paraffin embedding (FFPE) can alter the biochemical composition of tissues, potentially introducing spectral shifts, intensity variations, or additional peaks in Raman spectra. These changes may affect the direct comparison with fresh tissues. In this pilot study, however, the use of FFPE sections was necessary to ensure consistency with the histopathological workflow and to allow correlation with conventional stained slides. While our results confirm that meaningful spectral and morphological differences between healthy and malignant regions can still be observed, we recognize that FFPE processing represents a limitation for direct translational applications. Future work will focus on extending this multimodal approach to fresh, untreated tissues, thereby eliminating potential preparation-related artifacts and strengthening its clinical relevance.

## 4. Discussion

The first aim of this study was to perform a multidimensional analysis to investigate how Raman spectroscopy can capture the biochemical changes occurring in tissue regions that are morphologically distinguishable by AFM and SEM, and to correlate the outcomes from these techniques in order to extract diagnostic information from Raman data alone.

First of all the standard optical analysis revealed distinct tissue architectures, highlighting compact glandular structures, intertwined nerve fibers, and disorganized inflammatory and necrotic regions enabling the identification of areas of interest.

Subsequently Scanning Electron Microscopy imaging detailed microstructural variations, including tightly packed glandular cells, fibrous arrangements in nerves, and fragmented debris in necrotic zones. Atomic Force Microscopy further refined these observations by revealing nanoscale differences, such as surface compactness in glandular structures, porosity in inflammatory regions, and the dense proliferation of tumor cells.

The 813 *cm*^*-1*^ peak was consistently higher in nerve, blood vessel and stromal regions, which appeared porous and fibrous in AFM and also displayed organized structures under SEM. This band likely reflects collagen integrity and structural preservation in these morphologically healthy areas. Similarly, peaks between 920–956 *cm*^*-1*^, associated with proteins and lipids, were more prominent in nerves, stroma, and blood vessel, corresponding with the high organization seen in both AFM and SEM, suggesting active metabolic function and preserved extracellular architecture.

In contrast, tumor and necrotic areas, which displayed reduced porosity and irregular, compact topographies under AFM and SEM, were characterized by distinctive spectral changes. These included decreased intensities at 854–890 *cm*^*-1*^, indicating lipid and protein loss, a hallmark of cellular degradation.

Among the most reliable markers, the 1341 *cm*^*-1*^ peak (linked to nucleic acids) and the 1650 *cm*^*-1*^ peak (Amide I band) were elevated in tumor and necrotic tissues across multiple samples and patients, while, morphologically, these regions also showed dense cellularity, disrupted extracellular matrix, and evidence of necrosis, further reinforcing the diagnostic value of these bands.

Inflammatory regions, identified morphologically by their porous and irregular AFM profiles and ECM disorganization in SEM, showed reduced signals in the 1172–1268 *cm*^*-1*^ range, corresponding to Amide III and collagen components, supporting the observed degradation of structural proteins in these zones.

Interesting to note is that the signal from the gland exhibits the characteristics of both a compact tissue but with irregularities due to accumulation of extracellular matrix and cellular debris and a high presence of DNA a clear sign of malignancy.

Importantly, Raman spectroscopy not only reflected biochemical alterations but also captured features that were morphologically elusive. For example, regions with apparently intact tissue structure in AFM and SEM (like the gland) occasionally displayed Raman signatures of metabolic stress or early degeneration, suggesting that spectroscopy may provide earlier or more sensitive detection of pathological changes.

Taken together, these results demonstrate that distinct Raman bands can serve as molecular correlates of morphological features, enabling tissue classification even in the absence of imaging. This capability lays the groundwork for non-invasive diagnostic protocols where morphology informed Raman analysis can guide or even replace histopathological interpretation in certain contexts.

## 5. Conclusions

This pilot study first of all demonstrates the potential of a multimodal approach in which Raman spectroscopy, supported by SEM and AFM, is applied to cervical squamous cell carcinoma tissues to correlate the signal coming from morphologically distinguishable and undistinguishable regions.

Raman spectral features were found to correlate with micro and nanoscale morphological characteristics, enabling the differentiation of key tissue types such as glands, nerves, inflammatory infiltrates, necrotic zones, and malignant stroma. Notably, spectral markers such as those at 827 *cm*^*-1*^, 1341 *cm*^*-1*^, and 1650 *cm*^*-1*^ showed strong correspondence with pathological features like nucleic acid accumulation, protein degradation, and extracellular matrix remodeling offering robust indicators of malignancy and tissue degradation while spectral markers such as those at 813 *cm*^*-1*^, 920−956 *cm*^*-1*^ showed strong correspondence with the structural integrity of the tissue.

Importantly, the Raman analysis not only confirmed morphological observations but also revealed biochemical changes that were not always visible through imaging alone, suggesting, as already partially reported in literature [34–35], that Raman spectroscopy may serve as an early indicator of tissue transformation.

We acknowledge that the use of FFPE samples represents a limitation, as chemical fixation and paraffin embedding may alter Raman spectra and restrict the direct translation to diagnostic workflows. Nonetheless, this pilot work represents a stepping stone toward the future application of Raman spectroscopy on fresh, untreated tissues, where its non-destructive and label-free nature may offer significant benefits for intraoperative or ex vivo characterization.

Future studies, already underway, will extend this analysis to larger patient cohorts and incorporate multivariate statistical methods (e.g., PCA, LDA) to improve the robustness and automation of tissue classification. These advancements are expected to refine the diagnostic potential of Raman spectroscopy, establishing it as a clinically relevant tool for cervical cancer characterization and, eventually, for real-time assessment of surgical margins.

## Supporting information

S1 Fig Optical image of the first histological slide. (JPG)

S2 Fig Scanning Electron Microscopy images corresponding to the regions highlighted in Fig 2. Shown are (a, b) gland, (c, d) nerve, (e, f) central and (g, h) outer region of a blood vessel, presented here as zoomed-in details for higher-resolution visualization. (JPG)

S3 Fig Scanning Electron Microscopy images corresponding to the regions highlighted in Fig 2. Shown are (a, b) inflammatory infiltrates, (c, d) tumor tissue, (e, f) necrotic tissue and (g) stroma, presented here as zoomed-in details for higher-resolution visualization. (JPG)

S4 Fig Raman spectra of the support-slide of the sample. (PNG)

S5 Fig Averaged Raman spectra (50 spectra per tissue type) from nerve, tumor, necrosis, inflammatory infiltrate, blood vessel, gland, and stroma regions, normalized to the phenylalanine band at 1003 cm-1. The spectra are displayed separately to improve visual clarity and facilitate comparison among tissue components.
(PNG)

S6 Fig Box plots of Raman spectral intensities at selected peaks (813, 827, 875, 890, 920, 937, 956, 1030, 1081, 1172, 1240, 1268, 1294, 1316, 1338, 1379, 1420, 1450, 1485, 1605, 1615, 1655, and 1668 cm^-1^) across different histological regions (glands, tumor, stroma, inflammatory infiltrate, necrosis, nerves, and blood vessels). Note that all the Raman spectra in this comparison are normalized at 1 in 1087 cm^-1^.
(PNG)

S7 Fig Zoom in the various zones of the histological section. The red-marked area indicates the presence of tumor tissue.
(JPG)

S8 Fig Different Raman spectra, each one composed of the average of 200 cancerous or healthy cervical tissue, highlighting the spectral range of 950–1850 cm ^⁻ 1^ normalized to 1 (a.u.) at 1665 cm^⁻1^. The processed spectra reveal consistent differences between healthy and neoplastic areas, crucial for distinguishing tissue states.
(PNG)

S1 Data Fig 4B data.csv CSV file containing the numerical Raman spectral data corresponding to Fig 4B. The first column reports the Raman shift (cm-1), followed by the normalized averaged Raman intensities for each tissue type: nerve, gland, blood vessel, inflammatory infiltrate, necrosis, tumour, and stroma. Spectra are normalized to the phenylalanine band at 1003 cm-1 to allow direct comparison among tissue types.
(CSV)

S2 Data Fig 5 data.csv CSV file containing the numerical Raman spectral data corresponding to Fig 5. The first column reports the Raman shift (cm-1), followed by the averaged Raman spectra of tumour and healthy tissues. Additional columns report the minimum and maximum spectral intensity values at each Raman shift for tumour and healthy tissues, representing the variability range (min–max) calculated from 200 spectra per class. All spectra were baseline-corrected and normalized to the phenylalanine band at 1650 cm-1.
(CSV)

S3 Data S4 Fig data.csv CSV file containing the numerical Raman spectral data corresponding to S4 Fig. The first column reports the Raman shift (cm-1), while the second column reports the Raman intensity of the spectrum acquired from the support slide of the sample.
(CSV)

S4 Data S6 Fig data.csv CSV file containing the numerical Raman intensity data used to generate the box plots shown in S6 Fig. The first column reports the histological tissue type (glands, tumour, stroma, inflammatory infiltrate, necrosis, nerves, and blood vessels), followed by columns reporting the Raman spectral intensities at the selected peaks (813, 827, 875, 890, 920, 937, 956, 1030, 1081, 1172, 1240, 1268, 1294, 1316, 1338, 1379, 1420, 1450, 1485, 1605, 1615, 1655, and 1668 cm-1). All spectra were normalized to unity at 1087 cm-1 prior to intensity extraction.
(CSV)

S5 Data S8 Fig data.csv CSV file containing the numerical Raman spectral data corresponding to S8 Fig. The first column reports the Raman shift (cm-1), followed by columns reporting the averaged Raman intensities of healthy and cancerous cervical tissues. Each spectrum represents the average of 200 spectra per tissue class, extracted over the 950–1850 cm-1 spectral range and normalized to unity (a.u.) at 1665 cm-1.
(CSV)
