# Supplementary figures and images for: A pilot multimodal study of cervical cancer: Raman spectroscopy as a molecular fingerprint tool
Source: PLoS One. 2026 Jan 22;21(1):e0327286. doi: 10.1371/journal.pone.0327286 (PMC12826494; doi:10.1371/journal.pone.0327286)

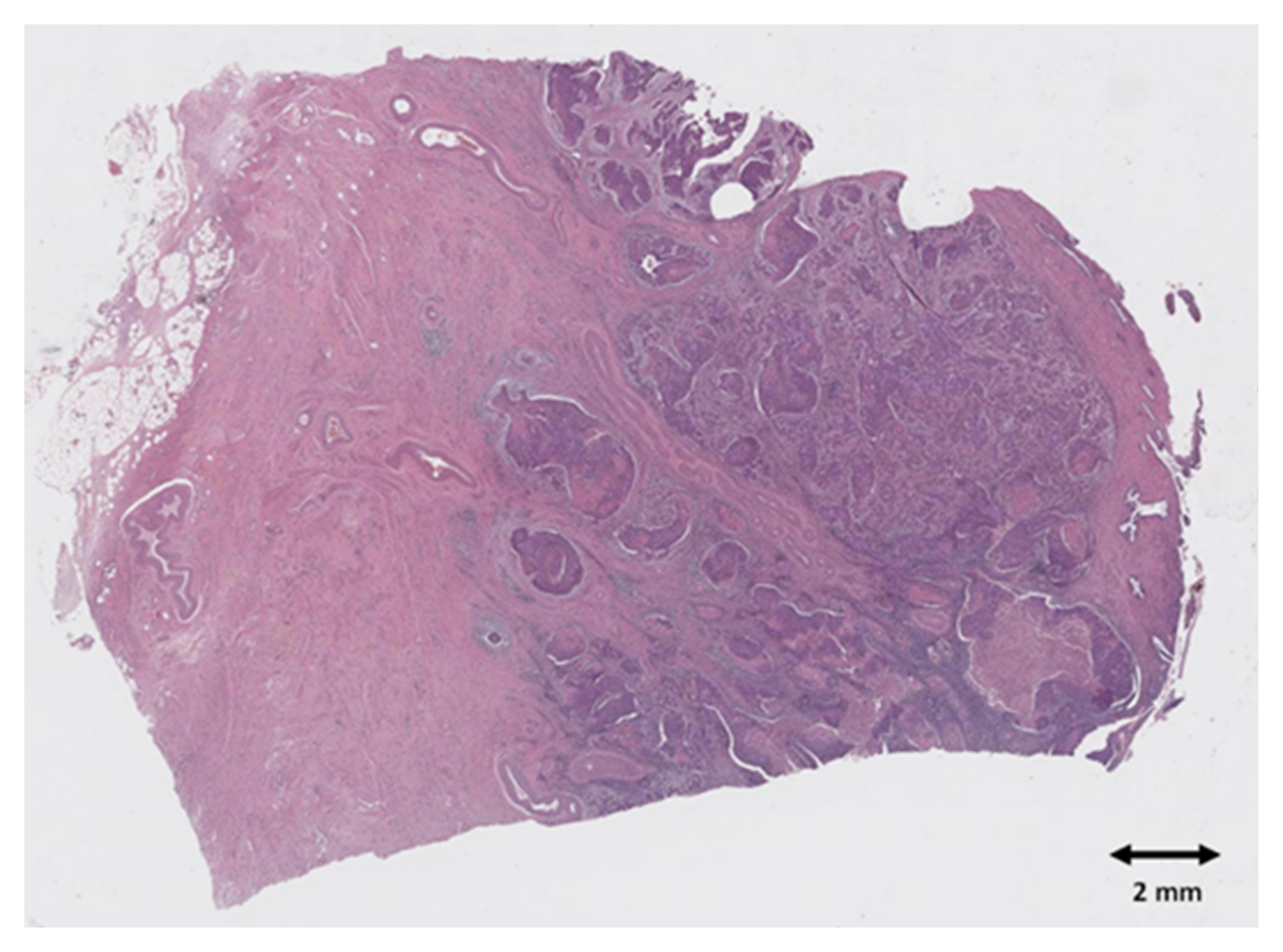

Supplement: S1 Fig — (JPG) [file pone.0327286.s001.jpg]

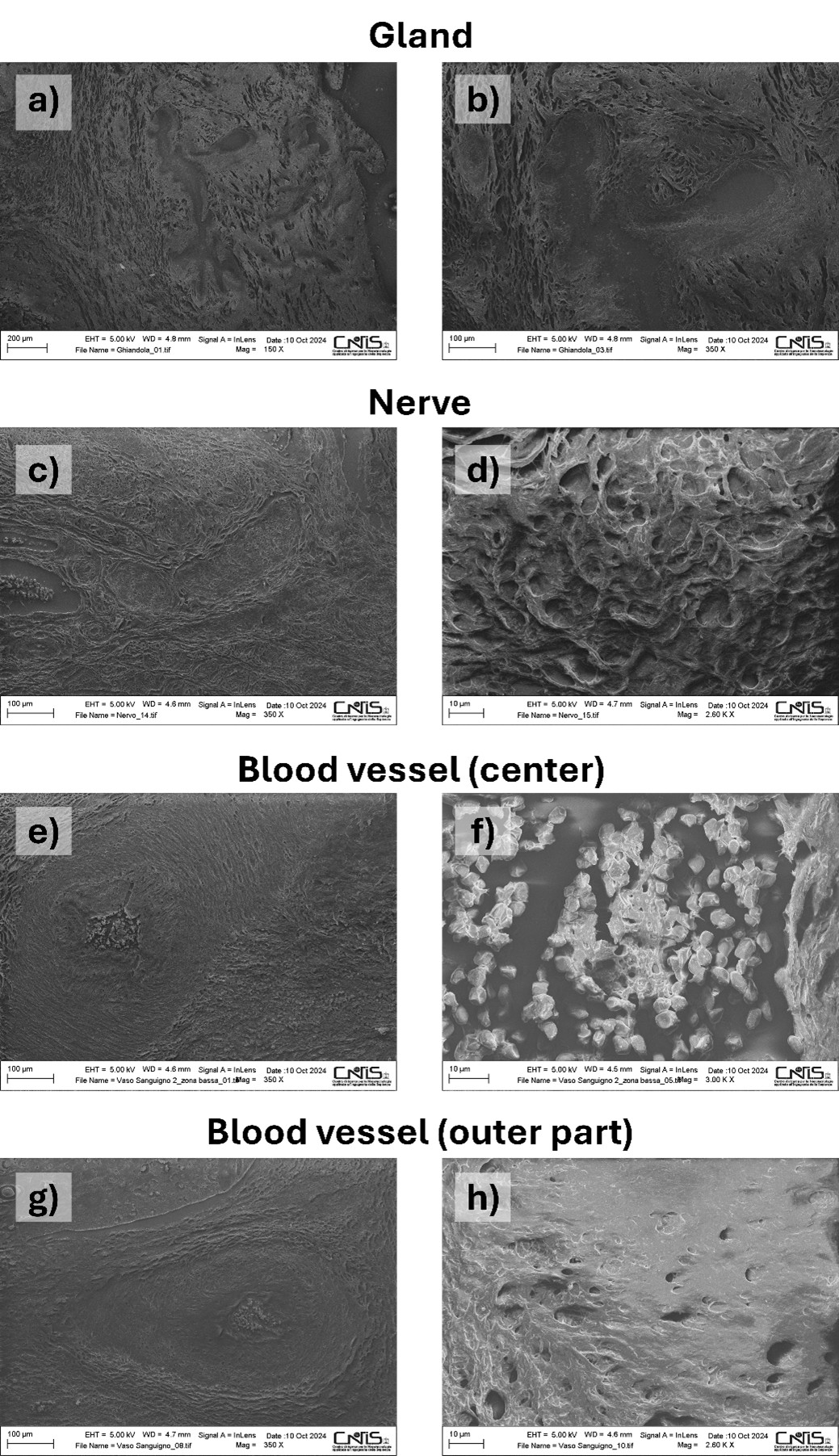

Supplement: S2 Fig — (JPG) [file pone.0327286.s002.jpg]

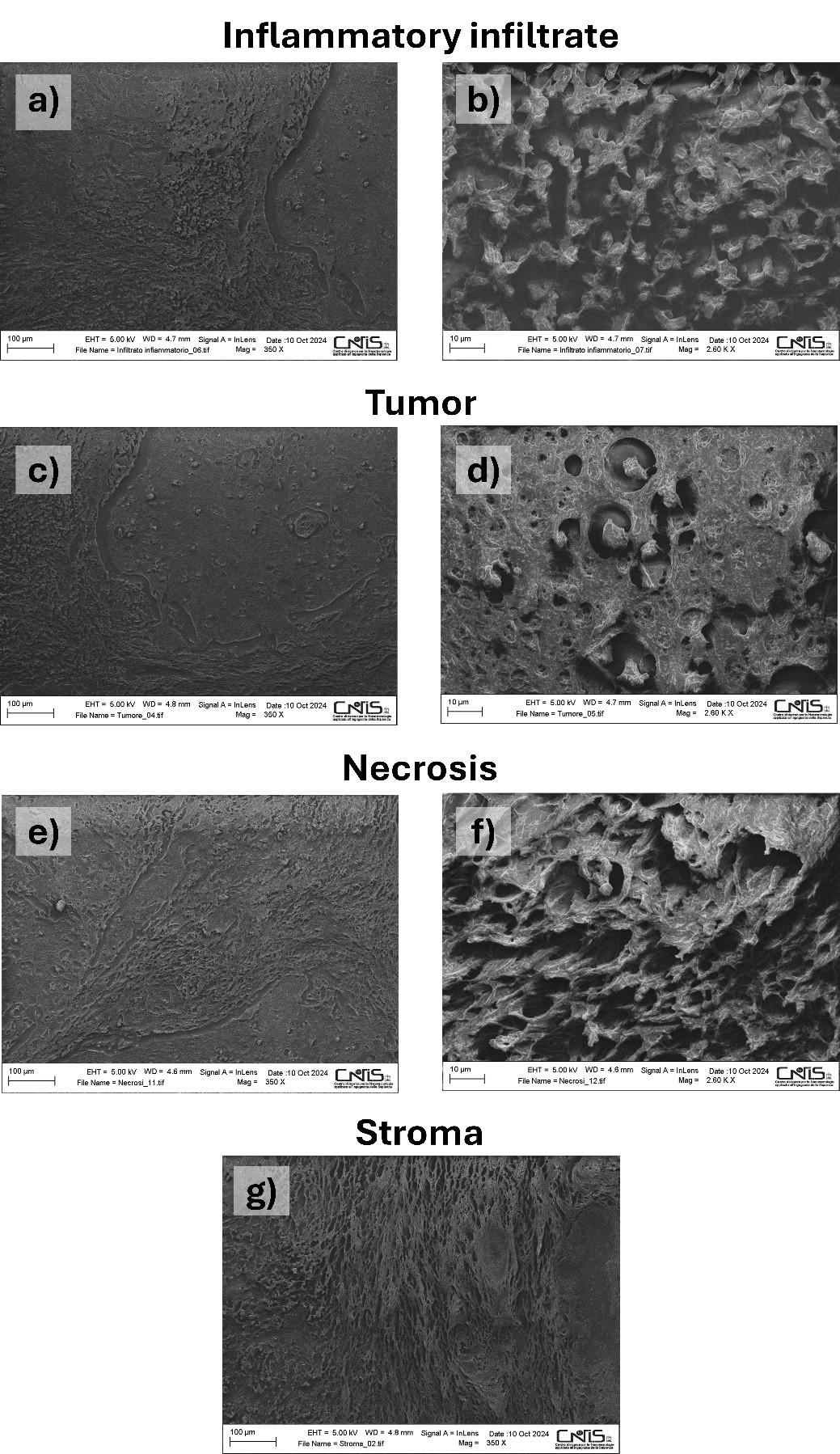

Supplement: S3 Fig — (JPG) [file pone.0327286.s003.jpg]

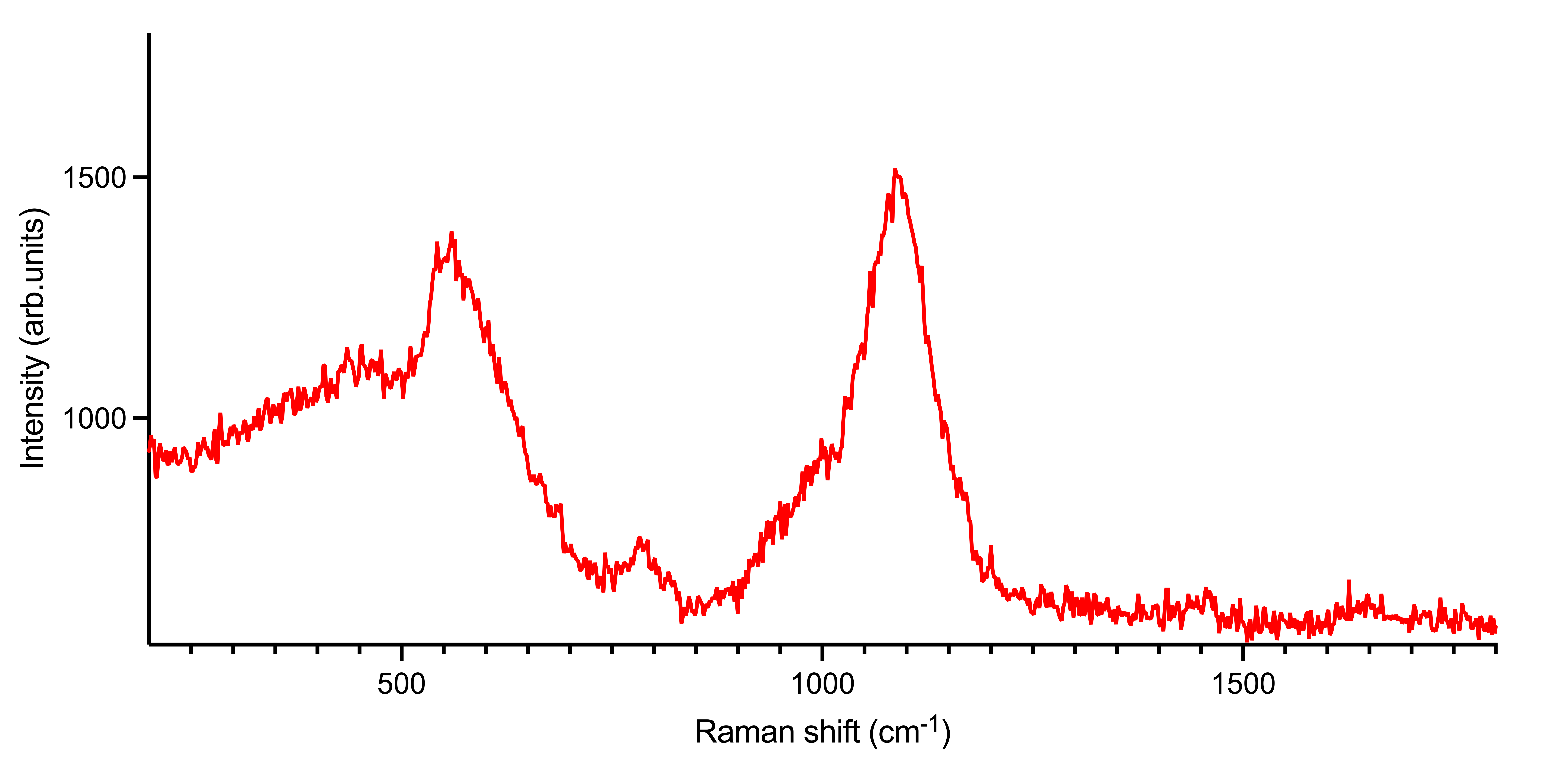

Supplement: S4 Fig — (PNG) [file pone.0327286.s004.png]

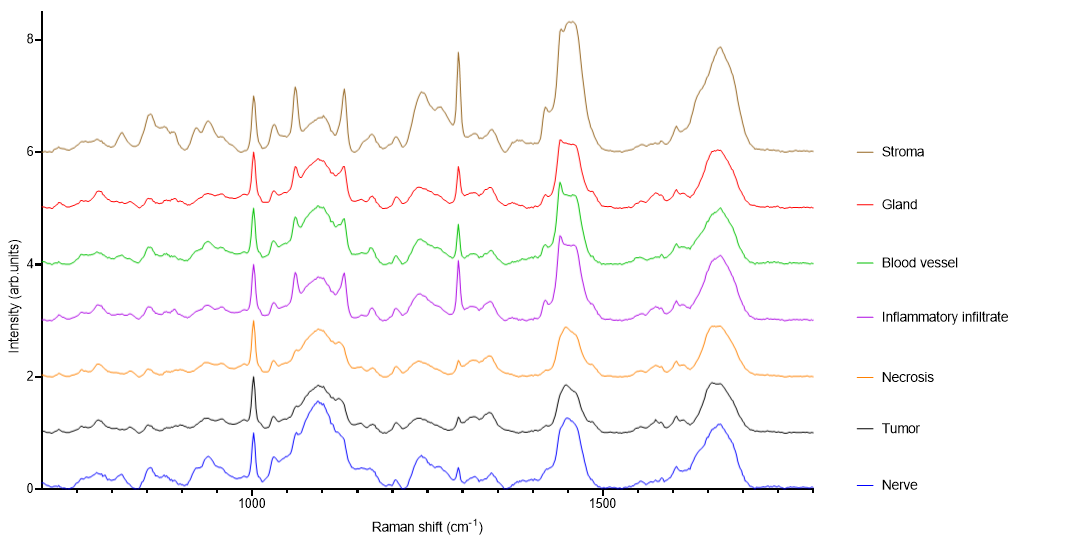

Supplement: S5 Fig — The spectra are displayed separately to improve visual clarity and facilitate comparison among tissue components. (PNG) [file pone.0327286.s005.png]

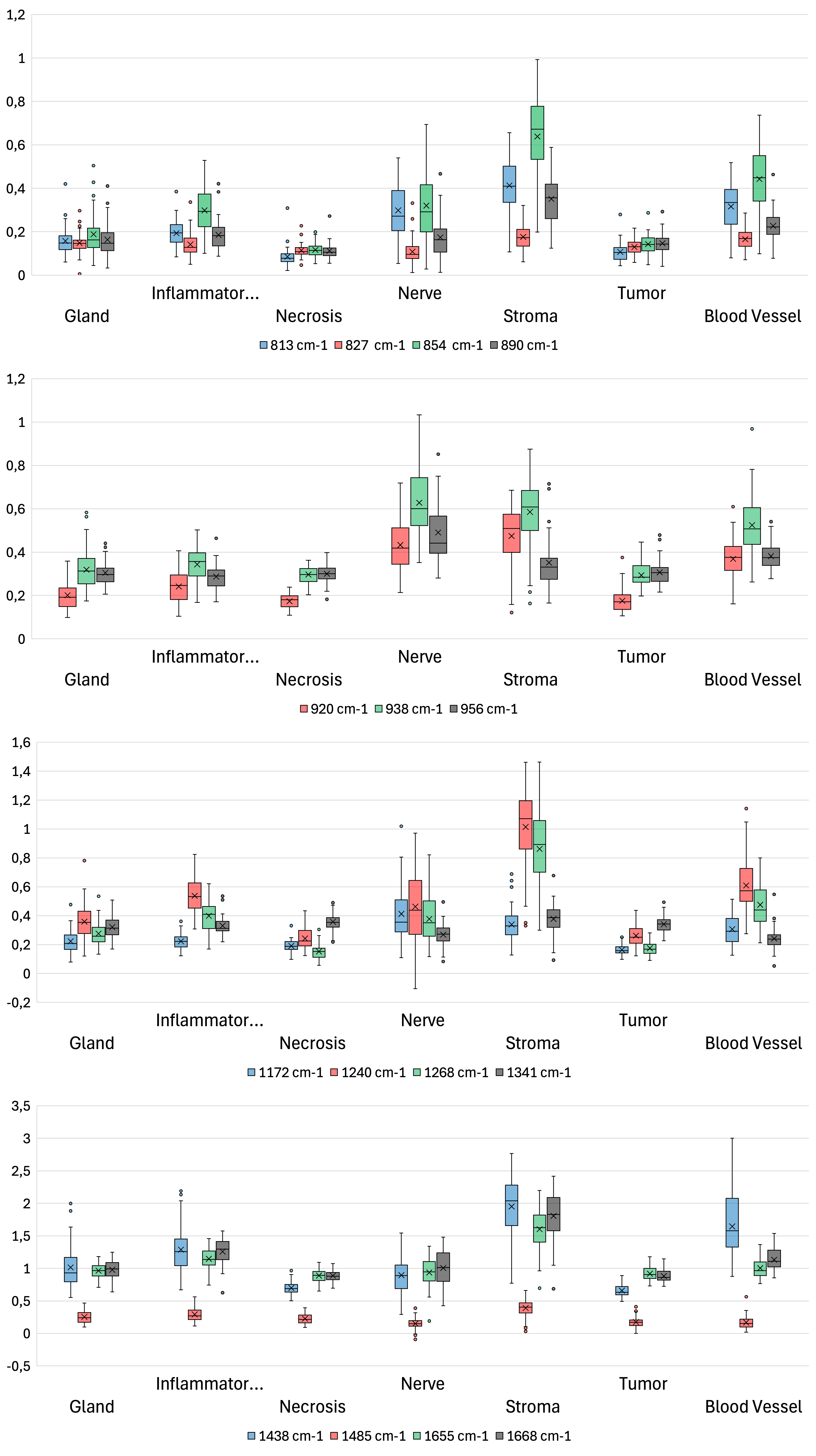

Supplement: S6 Fig — Note that all the Raman spectra in this comparison are normalized at 1 in 1087 cm-1. (PNG) [file pone.0327286.s006.png]

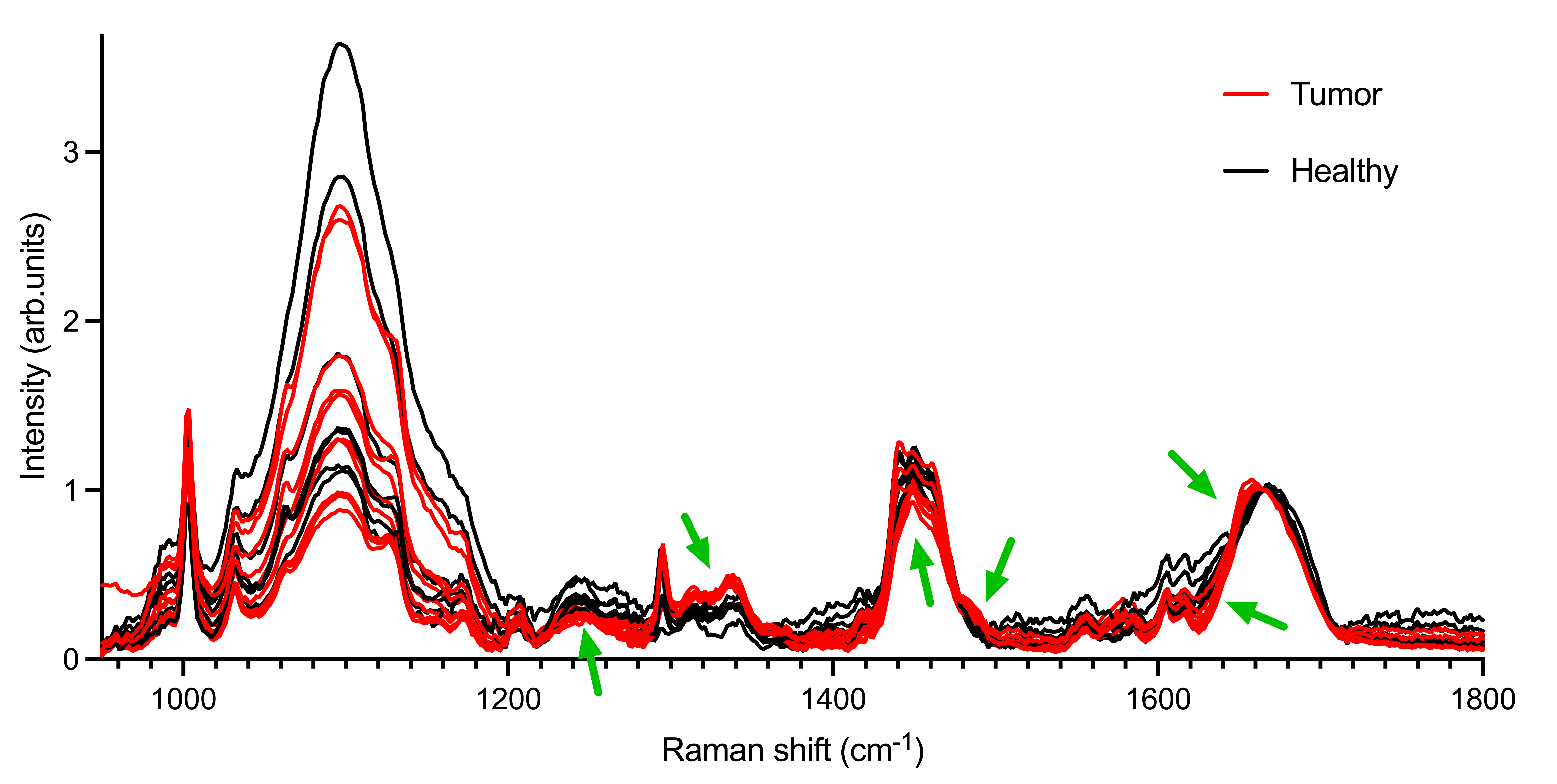

Supplement: S8 Fig — The processed spectra reveal consistent differences between healthy and neoplastic areas, crucial for distinguishing tissue states. (PNG) [file pone.0327286.s008.png]
